# Is the association between alcohol use and sickness absence modified by socioeconomic position? findings from the Stockholm public health cohort

**DOI:** 10.1186/s12889-023-16341-z

**Published:** 2023-08-04

**Authors:** Jonas Landberg, Emelie Thern

**Affiliations:** 1 Department of Public Health Sciences, Stockholm University, 106 91 Stockholm, Stockholm, Sweden; 2 Department of Clinical Neuroscience, Karolinska Institute, Stockholm, Sweden; 3 Institute of Environmental Medicine, Karolinska Institute, Stockholm, Sweden

**Keywords:** Health inequalities, Alcohol, Sickness absence, Lifestyle factors, Working conditions

## Abstract

**Background:**

The distribution of sickness absence tends to be socially patterned less is however known about the underlying mechanisms and pathways of the social gradient found in sickness absence. The present study aims to investigate (i) if the risk function between average volume of alcohol consumption and sickness absence is modified by socio-economic position (SEP), and (ii) whether such an effect modification can be attributed to differences in drinking patterns and other risk factors including other lifestyle behaviours, health status, and working conditions.

**Methods:**

The study was based on data from the Stockholm public health cohort 2006, with an analytical sample of 13 855 respondents aged 18–64 years. Self-reported information on occupational class (a measure of SEP), alcohol consumption, other lifestyle behaviour, health and working conditions was collected from the survey. The outcome of long-term (> 14 days) sickness absence between 2006 and 2008 was obtained from national registers. Negative binomial regression was used to estimate the Incidence Rate Ratios (IRR) with 95% confidence intervals (CI).

**Results:**

In the initial analyses, heavy drinking manual workers had a 5-fold increased risk of long-term sickness absence compared to non-manual employees who were moderate drinkers, and approximately 60% of the excess risk among heavy drinking manual workers was attributable to an interaction between alcohol use and SEP. Adjusting for working conditions was associated with the largest attenuation of the risk estimate, compared to other lifestyle behaviors and health. In the fully adjusted model, the IRR was further attenuated for the manual workers and the joint effect of SEP and heavy drinking remained in the final model with an attributable proportion of 49%.

**Conclusions:**

Individuals in Sweden with lower levels of SEP appear to be more vulnerable to alcohol consumption in relation to sickness absence, where differences in working conditions explained a large part but not all of the differential vulnerability.

## Introduction

Sickness absence is a major public health concern in Sweden and many other countries as it affects individuals, families, workplaces, and society at large with regards to well-being, productivity and finances. Approximately 4% of all Swedish employees were estimated to be on sick leave at any given time in 2012, a proportion that was more than twice as high as that of Denmark or Iceland [1]. Moreover, the cost of sickness absence was estimated at 66 billion Swedish crowns (corresponding to approximately 5.6 billion EUR) in 2021 [2]. The distribution of sickness absence tends to be socially patterned, like most health-related outcomes, with higher rates among groups with lower socioeconomic positions (SEP) [3–6]. Thus, to better tackle health inequalities further research is needed to better understand the underlying mechanisms and pathways of the social gradient found in sickness absence [3–6].

Alcohol use is a modifiable factor that has been linked to sickness absence, both short and long-term, in several studies [7–9]. Furthermore, considering that both hazardous alcohol use and related harm tend to be unequally distributed across SEP groups, with a higher prevalence among more disadvantaged groups, a plausible hypothesis would be that alcohol use contributes significantly to the social gradient in sickness absence [10, 11].

An increasing number of studies focusing on the social gradient in alcohol-related harm have assessed to what extent the gradient may be attributed to a differential exposure to hazardous levels and patterns of drinking across SEP-groups. According to a recent review, such SEP-differences in alcohol use account for up to 25% of the social gradient in alcohol-related morbidity and mortality [10]. Less research has assessed the contribution of differential exposure to alcohol use for the social gradient in sickness absence. However, a recent study found that around 20% of the SEP differences in long-term sickness absence (> 14 days), were accounted for by differential exposure to harmful levels and patterns of drinking across SEP-groups [12].

Another potential but less researched mechanism in this context is differential vulnerability [10]. This mechanism suggests that groups with low SEP are more likely to be exposed to multiple behavioral and social risk factors that may interact with their alcohol use, thereby resulting in an elevated risk for alcohol-related harm at a given level or pattern of drinking, in comparison to groups with higher socioeconomic backgrounds [10, 13, 14]. While there is increasing evidence that differential vulnerability constitutes an important mechanism underlying the social gradient in alcohol-related morbidity and mortality [10, 13, 15–17], less is known about the role of this mechanism in the context of SEP, alcohol use and sickness absence. Still, considering that important risk factors for sickness absence tend to cluster among low SEP, it is possible that they also would interact with alcohol use in low SEP groups, resulting in an increased risk of sickness absence from their drinking compared to groups from higher social strata. For instance, low SEP groups typically engage in more detrimental lifestyle habits, which may interact with alcohol consumption and increase the risk of alcohol-related health outcomes, including sickness absence [12, 14]. Further, groups with lower SEP tend to report poorer mental health and general well-being, also potentially intensifying the effects of alcohol consumption in this context [18, 19] Finally, a plausible assumption would be that a part of the differential vulnerability to a given level of alcohol use in relation to sickness absence could be attributed to an elevated prevalence of heavy episodic drinking (HED) among low SEP groups [10, 16].

The current study aims to increase our understanding of the social gradient in sickness absence by assessing whether there is a differential vulnerability to alcohol in relation to sickness absence across SEP groups. To this end, we will use the Stockholm Public Health Cohort (SPHC) to investigate if the risk function between the average volume of alcohol consumption and sickness absence is modified by SEP (measured by occupational class). To further strengthen our approach, we will also assess whether such an effect modification can be attributed to SEP differences in the frequency of HED and other risk factors including lifestyle behaviours, health status, and working conditions.

## Data and methods

### Study population

A register-based longitudinal cohort study based on the Stockholm Public Health Cohort (SPHC). The SPHC is a cross-sectional survey that is distributed every fourth year to an area- and sex-stratified random sample of residents in Stockholm County, Sweden. The self-reported data in the SPHC is regularly supplemented with information from various national registers, subsequently the data available in SPHC is extensive and contains a large amount of information on health, lifestyle, socio-economic and demographic factors. Further information on the data collection procedures, register linkages and response rate in the different waves of the SPHC is described in full elsewhere [20].

**Fig. 1:**
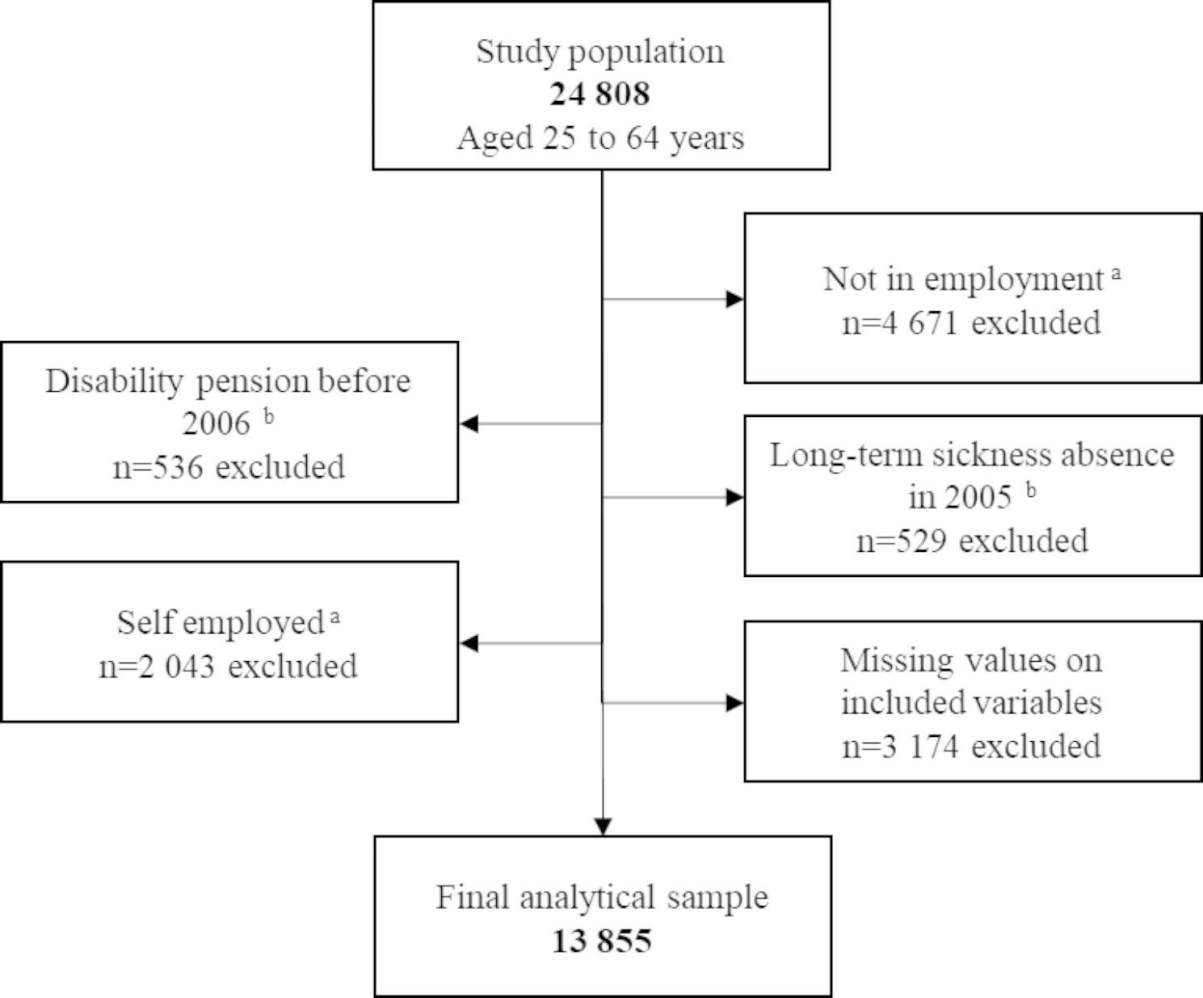
Flow chart describing the selection process of the participants ^a^ Self-reported information obtained from SPHC; ^b^ Information obtained from the Swedish National Social Insurance Registers (MiDAS)

For our study we used the baseline survey conducted in 2006 (n = 34 667; response rate 61%) [20], where the study population of interest was everyone aged between 25 and 64 years (n = 24 808). The analytical sample included all individuals between the age of 25 and 64 years who were employed, not on disability pension before 2006, without register based long-term sickness absence (> 14 days) in 2005 and complete information on the included variables (n = 13 855). See Fig. 1 for a flow chart over the selection process of the participants in the analytical sample. Individuals under the age of 25 years at baseline were excluded as their SEP is less established.

### Measures

#### SEP

SEP was defined using occupational class which was retrieved from the SPHC. In the survey respondents were asked to report their current occupation which was then classified into six groups according to the Swedish socioeconomic classification of occupations: (1) unskilled workers, (2) skilled workers, (3) lower non-manual employees, (4) intermediate non-manual employees, (5) higher non-manual employees and (6) self-employed [21]. In the main analyses, two groups of SEP were created: manual workers (including group 1 and 2) and non-manual employees (including group 3 to 5). Consequently, individuals who were defined as self-employed were excluded as it was difficult to determine if they were manual workers or non-manual employees.

#### Alcohol use

From the SPHC detailed information on self-reported average alcohol consumption was collected and defined as the average volume of weekly alcohol consumption during the past 12 months. Respondents were asked to specify the number of severings of different alcoholic beverages (i.e., wine, beer, cider, spirit) they would consume during a ‘normal week’, which was converted to grams of pure alcohol intake per week. For the main analyses, this was categorized into four different groups, with separate cut-off for men and women: abstainers (0 g), light (men and women: >0 to 84 g 100% alcohol/week), moderate (men: >84 to 252 g, women: >84 to 168 g), and heavy drinkers (men: >252 to 350 g, women: >168 to 280 g), which is in line with previous research [15, 22, 23].

#### Outcome: sickness absence

In the present study, the outcome of long-term sickness absence was defined as sickness absence episodes longer than 14 days. Information on long-term sickness absence was obtained from the Swedish National Social Insurance Registers (MiDAS) by record linkage to the SPHC. In Sweden the first 14 days of the sickness absence spell is covered by the employer. If the individual is still on sickness absence after day 14 then the Social Insurance Agency covers the costs, and the information is registered in the social insurance registers. Consequently, the information in MiDAS includes the number of sickness absence days after day 14 of the sickness absence spell. As outcome, we thus counted the number of sickness absence days (after day 14) that occurred from the date of entering the study (14 September 2006 at the earliest) until date of emigration (information obtained from Statistics Sweden, SCB), retirement by age (obtained from SCB), disability pension (obtained from the MiDAS register), death (obtained from the Swedish Cause of Death Register) or end of follow-up, December 31, 2008.

#### Explanatory variables

Information on common risk factors for sickness absence that tend to be more prevalent among lower SEP-groups were obtained from the SPHC. Likewise, information on the background covariates sex and age were obtained from SPHC.

*HED*: Self-reported information on the frequency of heavy episodic drinking (HED) was included and defined as the number of occasions respondents consumed 120 g of alcohol (100% pure alcohol) or more during the last 12 months. This is equivalent to a half-bottle of spirits, two bottles of wine or six cans of strong beer. Similar to previous research, the responses were divided into five categories: abstainers, alcohol consumers with no HED, HED one to six times per year, HED one to three times per month and HED at least once a week [24] .

*Lifestyle factors*: In line with previous research, we created a lifestyle index to assess the possible contribution of several lifestyle factors: smoking, snuff use, BMI, physical activity and sleep, as well as fruit and vegetable intake [15, 24–26]. For each unhealthy lifestyle factor the individual would receive one point: current daily smoker, current daily use of snuff, being underweight or overweight/obese according to BMI, performing less than 150 min of moderate PA per week, sleeping more or less than 7–9 h, or eating less than four portions of fruit and vegetable per day. A higher score indicated an unhealthier lifestyle. For the main analyses the lifestyle index scores were categorised into three groups: most healthy (0–2 points), moderately healthy (3–4 points) and most unhealthy (5–6 points).

##### Health

Two measures of health were obtained from the SPHC survey; mental health and health-related quality of life. The 12-item version of the General Health Questionnaire (GHQ12), a well-established scale for screening of psychiatric morbidity in the general population, was used to measure mental health [27]. In line with previous research, a 3/4 threshold was applied to identify cases with poor mental health status [24, 28]. The EQ-5D instrument was used to measure health-related quality of life (HRQoL) [29]. The measure specifies five dimensions; mobility (ability to walk), self-care (daily personal care), usual activities (e.g., work, study, housework, family, or leisure activities), pain/discomfort and anxiety/depression. Individuals were asked to respond ‘no problems’, ‘some problems’ or ‘severe problems’ to each of the five dimensions. In the main analyses, ‘some problems’ and ‘severe problems ‘ were collapsed into one category, which has been done previously [15, 24].

*Working conditions*: Information on physical and psychosocial working conditions were used as measures of working conditions and obtained from SPHC. For *physical work conditions*, individuals were asked to report to what extent they moved or exerted themself physically at work during the past 12 months: sedentary work (your job is predominantly sedentary); light physical work (your work involves moving/walking around a lot, but no heavy lifts); moderately heavy work (your work involves moving/walking around a lot, some heavy lifts, and/or walking on stairs or slopes); and heavy work (your work involves lifting heavy objects and exerting a lot of physical effort). For *psychosocial working conditions*, individuals were asked to respond to eight questions based on the demand-control model with regards to the degree of job control and job demands (see [30] for a more detailed description of the questions). In line with previous research, the eight questions were combined to create four mutually exclusive groups; high strain (low control with high demands); passive work (low control with low demands); active work (high control with high demands); and low strain (high control with low demands) [24].

### Statistical analyses

The association between the joint effect of SEP and alcohol consumption and sickness absence was estimated using Generalized linear model (GLM) regression. Since the distribution of the outcome was over-dispersed, we applied Negative binomial regression (rather than Poisson regression). The estimates are expressed as Incidence Rate Ratios (IRR) with 95% confidence intervals (CI). An offset variable measuring the logarithm of person–time at risk was included in the analyses, calculated from the date a participant entered the study (14 September 2006 at the earliest) until being censored by date of emigration, retirement by age, disability pension, death, or end of follow-up (31 December 2008). In accordance to SPHC recommendation, calibrated weights computed by Statistics Sweden using register-based auxiliary data from the Total Population Register on age, sex, country of birth, area of residence, civil status, income, educational level and sickness allowance, was employed to reduce non-response error [20].

To test if the association between average volume of alcohol consumption and sickness absence is modified by SEP, we constructed a joint exposure variable that stratified the four consumption groups by SEP (resulting in eight categories), which has been done previously [15, 24]. To assess the presence of an additive interaction of alcohol consumption and SEP in relation to the outcome we calculated the relative excess risk due to interaction (RERI) (RERI = IRR_11_-IRR_10_-IRR_01_ + 1). This measure estimates to what degree the IRR of the combined exposures of being both a heavy drinker and a manual worker (IRR_11_) is larger (or smaller) than the sum of the IRR of the two exposures considered individually, i.e. being a heavy drinker/non manual-employee (IRR_10_) or being a moderate drinker/manual worker (IRR_01_). Using the Delta method 95% CI:s were calculated [31] as well as the attributable proportion due to the interaction (AP = RERI/IRR_11_). Following recommendations, we present our interaction analyses with a single common reference group [31]; non-manual employees with moderate drinking as reference group.

First, adjustment for the background covariates age (continuous) and sex was performed (Model 1). Then a series of models with adjustments for background covariates and separate adjustments for (i) frequency of HED, (ii) lifestyle factors and health status and (iii) working conditions were performed. In the final model all covariates were entered simultaneously. All analyses were performed using Stata Statistical Software, release 15.

## Results

### Descriptive

Table 1 shows the baseline characteristics of the study population. A larger proportion, 74%, of all respondents were defined as non-manual employees. The prevalence of heavy drinkers was similar among manual workers and non-manual employees. However, HED ≤ 3 times/month and HED ≥ once a week were more common among manual workers. Moreover, manual workers reported less healthy lifestyle habits, lower HRQoL, and worse working conditions compared to non-manual employees. It should be mentioned, however, that mental health status formed an exception, with a higher prevalence of poor mental health among non-manual employees, compared to manual workers.

**Table 1:** Baseline characteristics of the study sample

	Manual workers			Non-manual employees				Total	
	*n*	*%*		*n*		*%*		*n*	*%*
**n**	3645	26.3		10,210		73.7		13,855	100
**Sex** (% males)	2125	58.3		4969		48.7		7094	51.2
**Age (mean)**		42.9				42.8			42.8
**Nr sickness absence days (mean)**		9.3				4.6		5.8	
**Volume of consumption**									
Abstainers	544	14.9		560		5.5		1104	8.0
Light drinkers	1643	45.1		4808		47.1		6451	46.6
Moderate drinkers	1054	28.9		3804		37.3		4859	35.1
Heavy drinkers	404	11.1		1038		10.2		1442	10.4
**Frequency of HED**									
Abstainers	544	14.9		560		5.5		1104	8.0
Drinkers with no HED	1179	32.3		5108		50.0		6287	45.4
HED 1–6 times/year	1012	27.8		2872		28.1		3884	28.0
HED ≤ 3 times/month	609	16.7		1273		12.5		1883	13.6
HED > once a week	301	8.3		396		3.9		698	5.0
**Lifestyle**									
Most healthy	1562	42.9		5235		51.3		6796	49.1
Moderately healthy	1821	50.0		4521		44.3		6341	45.8
Less healthy	262	7.2		454		4.5		716	5.2
**Poor mental health (GHQ-12)** ^a^	401	11.0		1294		12.7		1694	12.2
**Health related quality of life (Eq. 5d)**									
Some problems or more	2046	56.1		4543		44.5		6589	47.6
**Physical working conditions**									
Sedentary or light	1177	32.3		9016		88.3		10,195	73.6
Moderate	1551	42.6		1050		10.3		2601	18.8
Heavy	917	25.2		144		1.4		1060	7.7
**Psychosocial working conditions**									
High strain	1127	30.9		1500		14.7		2627	19.0
Active	1090	29.9		5382		52.7		6472	46.7
Passive	607	16.6		673		6.6		1279	9.2
Low strain	822	22.6		2656		26.0		3475	25.1

HED: Heavy episodic drinker. ^a^ a 3/4 threshold was applied to identify cases with poor mental health status

### Bivariate associations with sickness absence for each exposure and explanatory variable

Table 2 shows the single effects of the exposure variables SEP and alcohol use, as well as the bivariate association with sickness absence for each explanatory variable (all models adjusted for sex and age). A u-shaped association between average volume of alcohol consumption and sickness absence was found, with increased rates of sickness absence among heavy drinkers and abstainers relative to moderate drinkers. Compared to non-manual employees, manual workers had more than two times higher rates of sickness absence. For each explanatory variable, individuals belonging to the most unfavorable category evidenced a significantly higher rate of sickness absence compared to the reference group.

**Table 2:** Negative binomial regression models of the association between each exposure/covariate and sickness absence. Incidence Rate Ratios (IRR) and 95% confidence intervals (CIs). All models adjusted for sex and age

	IRR	95% CI	
***Single effects of exposures***			
**Average volume of consumption**			
Abstainers	2.20	1.56	3.10
Light drinkers	1.27	0.98	1.64
Moderate drinkers (ref)			
Heavy drinkers	1.68	1.16	2.42
**SEP**			
Manual workers	2.32	1.82	2.97
Non-manual employees (ref)	1.00		
***Covariates***			
**Frequency of HED**			
Abstainers	2.09	1.44	3.05
Drinkers with no HED	1.09	0.84	1.42
HED 1–6 times/year (ref)	1.00		
HED ≤ 3 times/month	1.35	0.82	2.24
HED > once a week	2.38	1.47	3.87
**Lifestyle**			
Most healthy (ref)	1.00		
Moderately healthy	1.46	1.17	1.83
Less healthy	1.52	1.01	2.26
**Poor mental health (GHQ-12)**	2.24	1.60	3.13
**Health related quality of life (Eq. 5d), some problems or more**	2.95	2.36	3.71
**Physical working conditions**			
Sedentary or light (ref)	1.00		
Moderate	2.12	1.67	2.68
Heavy	3.36	2.20	5.13
**Psychosocial working conditions**			
Low strain (ref)	1.00		
Active	1.07	0.82	1.41
Passive	1.61	1.11	2.34
High strain	1.85	1.34	2.56

HED: Heavy episodic drinking

### Estimation of the joint effect between SEP and alcohol consumption on sickness absence

Table 3 shows the estimated associations between the joint exposure variable and sickness absence. After basic adjustments (sex and age), manual workers that were light to heavy drinkers, had approximately 2 to 5 times higher rates of sickness absence, compared to the reference group non-manual employees who were moderate drinkers. Conversely, heavy drinking non-manual employees did not differ significantly from the reference group. Calculation of RERI, revealed a significant joint effect of SEP and heavy drinking (RERI: 2.86, 95% CI 0.52 to 5.20) with an AP of 58, implying that approximately 60% of the excess risk among heavy drinking manual workers was attributable to an interaction between average volume of alcohol consumption and SEP.

**Table 3:** Negative binomial regression models of the the joint effect between socioeconomic position and average volume of alcohol consumption on the outcome of sickness absence. Incidence Rate Ratios (IRR) and 95% confidence intervals (CIs).

	Non-manual empl.			Manual workers			Tests of interaction		
	IRR	95% CI		IRR	95% CI		RERI ^a^(AP ^a^ )	95% CI	
**Model 1**									
Abstainer	2.18	1.27	3.74	3.03	1.96	4.69			
Light drinker	1.21	0.89	1.63	2.36	1.55	3.61			
Moderate drinker	1.00 (ref)			2.14	1.47	3.13			
Heavy drinker	0.97	0.60	1.56	4.97	3.04	8.13	2.86 (58)	0.52	5.20
**Model 2**									
Abstainer	2.29	1.26	4.13	3.17	1.94	5.19			
Light drinker	1.21	0.89	1.64	2.33	1.57	3.45			
Moderate drinker	1.00 (ref)			2.07	1.42	3.01			
Heavy drinker	0.90	0.54	1.51	4.05	2.51	6.55	2.08 (51)	0.24	3.92
**Model 3**									
Abstainer	2.41	1.34	4.31	3.31	2.04	5.38			
Light drinker	1.23	0.90	1.68	2.08	1.43	3.00			
Moderate drinker	1.00 (ref)			1.93	1.33	2.79			
Heavy drinker	0.85	0.52	1.39	4.41	2.49	7.81	2.63 (60)	0.23	5.03
**Model 4**									
Abstainer	2.17	1.26	3.71	2.13	1.27	3.59			
Light drinker	1.25	0.92	1.70	1.46	0.99	2.17			
Moderate drinker	1.00 (ref)			1.41	0.95	2.09			
Heavy drinker	0.99	0.63	1.56	3.04	1.77	5.22	1.64 (54)	0.09	3.20
**Model 5**									
Abstainer	2.46	1.30	4.67	2.53	1.45	4.39			
Light drinker	1.26	0.92	1.72	1.46	0.97	2.18			
Moderate drinker	1.00 (ref)			1.36	0.91	2.03			
Heavy drinker	0.80	0.49	1.30	2.29	1.33	3.92	1.12 (49)	0.01	2.24

Model 1: Joint exposure variable (with eight categories) adjusted for sex and age (continuous)

Model 2: Additional adjustment for frequency of heavy episodic drinking (HED)

Model 3: Additional adjustment for lifestyle index, health related quality of life (Eq. 5D) and mental health (GHQ12)

Model 4: Additional adjustment, for physical and psychosocial working conditions

Model 5: Fully adjusted

^**a**^ Relative excess risk due to interaction (RERI = IRR_11_-IRR_10_-IRR_01_ + 1)

^**b**^ Attributable proportion due to interaction (AP = RERI/IRR_11_)

Separate adjustments for frequency of HED (Model 2), as well as for lifestyle and health status (Model 3), only resulted in marginal attenuations of the IRRs. Moreover, adjustment for working conditions (Model 4) resulted in larger attenuations, yet the increased risk among heavy drinking manual workers remained. In the fully adjusted model, the IRR:s were further attenuated for the manual workers (by approximately 70% compared to Model 1). Still, the joint effect of SEP and heavy drinking remained in the final model with an AP of 49%.

## Discussion

The result of this study suggests that SEP modifies the association between average volume of alcohol consumption and sickness absence, such that at any given level of alcohol use individuals with lower SEP had higher rates of sickness absence. Differential distribution of HED and work conditions explain a large part but not all of the differential vulnerability, subsequently the effect modification of alcohol use by SEP remained.

The current results extend and further support previous research on the social gradient in alcohol-related harm [13, 15, 16]. In contrast to a recently published study from Norway, we found that the same levels of drinking were associated with higher rates of sickness absence in groups with lower compared to higher SEP [32]. Specifically, all patterns of drinking among the manual workers were associated with an increased risk of sickness absence when compared to non-manual employees with moderate drinking. It is notable that the association between average volume and sickness absence, foremost appears to apply to manual workers, considering that heavy drinking was not associated with higher rates of sickness absence compared to moderate drinkers among non-manual employees. The highest IRR was found for heavy drinking manual workers who had approximately five times higher rates of sickness absence compared to the reference group. Calculations of RERI revealed that approximately 60% of the excess risk among heavy drinking manual workers was attributable to the interaction between alcohol use and SEP.

To address our second aim, we adjusted the main association for several measures of other lifestyle factors, health, and factors related to sickness absence such as physical and psychosocial work conditions [5, 33]. In line with previous research on mortality and alcohol-related morbidity and mortality we found that the joint effect of alcohol consumption and SEP could not be attributed to differences in HED, other lifestyle factors, and health [13, 15, 16]. Work conditions appear to explain a substantial but not all the joint effect, subsequently SEP remained a modifying factor. In contrast to previous findings for more alcohol specific outcomes, such as alcohol-related morbidity and mortality, the differential vulnerability was explained by other factors to a smaller extent [13, 15, 16]. A potential reason for this is that sickness absence is a much more complex outcome, relating to reduced work ability usually linked to ill health as well as loss of work-related income. Consequently, there could be other underlying mechanisms with regard to sickness absence that might explain that the social gradient such as differential consequences [34]. This mechanism suggests that individuals with higher SEP might be more capable to deal with the direct cost of health care and income forgone, as well as being an occupation that can better hide alcohol-related absenteeism, and adapt the current work conditions to prevent new spells of sickness absence [8]. In other words, although having the same level of alcohol-related health problems, it might be more difficult to manage one’s job within a low SEP occupation. Moreover, there could potentially be a healthy worker effect as individuals with consistent harmful alcohol use which causes health problems might be selected out of the labour market to a higher degree, implying that the role of alcohol for the social gradient in alcohol-related absenteeism probably is larger than estimated by our current analyses [35]. Furthermore, the results of the current study demonstrated that differences in physical and psychosocial work explained a large of the social gradient found in sickness absence, which seems reasonable as some occupations are more mentally and physically challenging than others [8]. Finally, it is notable that abstainers had similar rates of sickness absence among both SEP-groups in the fully adjusted model, implying that the mechanisms underlying the increased sickness absence rates among this drinking group probably is less socially patterned.

To better tackle health inequalities in health in general and sickness absence in particular, an increased understanding of how an individual’s social position influence their risk of ill health is vital [36]. In line with previous research, the current study found that the social gradient in sickness absence is not only related to differential exposure of alcohol but also due to the differential vulnerability to the negative effects of alcohol [15, 36]. Consequently, universal alcohol policy measures that effectively help reduce the total alcohol consumption will most likely have a larger impact on sickness absence among groups with low SEP, as they are the most vulnerable - subsequently reducing the social gradient of this outcome [36, 37]. Furthermore, previous research on an aggregated level suggests that deceased total alcohol consumption can lead to a reduction in sickness absence costs [38, 39].

### Strengths and limitations

Major strengths of the current study include being able to prospectively follow a large cohort, detailed information on exposure before the outcome, and several known risk factors. Unfortunately, we were however only able to include one measurement point of alcohol consumption which is a limitation as alcohol consumption tend to change during the life course [40]. Furthermore, there could be a difference in adjusting harmful alcohol consumption prior to sickness absence, where higher SEP groups a more likely to adjust their consumption downward [41]. Furthermore, including register-based information on the outcome of sickness absence ensures nationwide coverage and complete follow-up information. Several reviews have, however, shown that the effects of alcohol on sickness absence are found when sickness absence is based on register data and not to the same extent when the information is self-reported [9, 42].

Further strengths include being able to include extensive information on other risk-factors that could potentially explain the differential vulnerability to alcohol in relation to sickness absence among SEP groups as this has not been extensively done in previous research [8].

Due to power issues, we were unable to stratify the analyses by sex which could be seen as a limitation as drinking behaviour and sickness absence differs between males and females, where males consume large quantities of alcohol and females tend to be overrepresented among sickness absence beneficiaries [43, 44]. Previous research has found that increased alcohol use increase the risk of sickness absence among both men and women, where some evidence suggests this association could be stronger for females compared to males [8, 9].

There is most likely an under-representation of heavy and problem drinkers in the sample, suggesting that the association between alcohol use and sickness absence might be underestimated to some extent. It should also be acknowledged that the threshold for HED in this study, set at 120 g on a single occasion, is higher than the standard cut-off. It is possible that using a lower threshold, such as 60 g, could lead to a larger attenuation of the main association. Finally, alcohol use and other explanatory factors were only assessed at baseline. Previous research indicates that accounting for time-varying changes in health behaviors tend to yield larger estimates of their role for socioeconomic inequalities in health [41, 45].

## Conclusion

In conclusion, our findings add support to the notion that the mechanism of differential vulnerability to average alcohol consumption by SEP, is also valid for sickness absence – so that the same level of drinking is associated with higher rates of sickness absence in lower compared to higher SEP-groups. While this partly could be attributed to differences in working conditions. It did, together with lifestyle and working conditions, not fully explain the effect joint effect among heavy drinkers that are manual workers.

## Data Availability

The data that support the findings of this study are available from the Centre for Epidemiology and Community Medicine at Stockholm County Council, but restrictions apply to the availability of these data, which were used under license for the current study, and so are not publicly available. Data are however available from the Centre for Epidemiology and Community Medicine at Stockholm County Council (email: sphc.ces.slso@sll.se).

## Abbreviations

AP: Attributable portion
BMI: Body mass index
HED: Heavy episodic drinking
HRQoL: Health-related quality of life
IRR: Incidence Rate Ratios
MiDAS: Swedish National Social Insurance Registers
SEP: Socioeconomic position
SPHC: Stockholm Public Health Cohort
PA: Physical Activity
RERI: Relative excess risk due to interaction
